# Natural Products/Bioactive Compounds as a Source of Anticancer Drugs

**DOI:** 10.3390/cancers14246203

**Published:** 2022-12-15

**Authors:** Syeda Tasmia Asma, Ulas Acaroz, Kálmán Imre, Adriana Morar, Syed Rizwan Ali Shah, Syed Zajif Hussain, Damla Arslan-Acaroz, Hayri Demirbas, Zehra Hajrulai-Musliu, Fatih Ramazan Istanbullugil, Ali Soleimanzadeh, Dmitry Morozov, Kui Zhu, Viorel Herman, Abdelhanine Ayad, Christos Athanassiou, Sinan Ince

**Affiliations:** 1 Department of Food Hygiene and Technology, Faculty of Veterinary Medicine, Afyon Kocatepe University, Afyonkarahisar 03200, Turkey; 2 ACR Bio Food and Biochemistry Research and Development, Afyonkarahisar 03200, Turkey; 3 Department of Animal Production and Veterinary Public Health, Faculty of Veterinary Medicine, University of Life Sciences “King Mihai I” from Timișoara, 300645 Timisoara, Romania; 4 Department of Animal Nutrition and Nutritional Diseases, Faculty of Veterinary Medicine, Afyon Kocatepe University, Afyonkarahisar 03200, Turkey; 5 Department of Chemistry and Chemical Engineering, SBA School of Science & Engineering (SBASSE), Lahore University of Management Sciences (LUMS), Lahore 54792, Pakistan; 6 Department of Biochemistry, Faculty of Veterinary Medicine, Afyon Kocatepe University, Afyonkarahisar 03200, Turkey; 7 Department of Neurology, Faculty of Medicine, Afyonkarahisar Health Sciences University, Afyonkarahisar 03030, Turkey; 8 Department of Chemistry, Faculty of Veterinary Medicine, Ss. Cyril and Methodius University of Skopje, 1000 Skopje, North Macedonia; 9 Department of Chemistry and Technology, Faculty of Veterinary Medicine, Kyrgyz-Turkish Manas University, Bishkek KG-720038, Kyrgyzstan; 10 Department of Theriogenology, Faculty of Veterinary Medicine, Urmia University, Urmia 5756151818, Iran; 11 Department of Epizootology and Infectious Diseases, Vitebsk State Academy of Veterinary Medicine, 210026 Vitebsk, Belarus; 12 National Center for Veterinary Drug Safety Evaluation, College of Veterinary Medicine, China Agricultural University, Beijing 100193, China; 13 Department of Infectious Disease and Preventive Medicine, Faculty of Veterinary Medicine, University of Life Sciences “King Mihai I” from Timișoara, 300645 Timisoara, Romania; 14 Department of Physical Biology and Chemistry, Faculty of Nature and Life Sciences, Université de Bejaia, Bejaia 06000, Algeria; 15 Laboratory of Entomology and Agriculture Zoology, Department of Agriculture, Crop Production and Rural Environment, University of Thessaly, 38446 Volos, Greece; 16 Department of Pharmacology and Toxicology, Faculty of Veterinary Medicine, Afyon Kocatepe University, Afyonkarahisar 03200, Turkey

**Keywords:** natural products, anticancer drugs, medicinal plants, medicinal mushrooms

## Abstract

**Simple Summary:**

Cancer is considered as a large group of diseases involving abnormal cell growth, which results in an alarming rise in the mortality rate at the worldwide level. Presently, the main point of interest in pharmaceutical research is the development of the novel and efficient anticancer drugs based on natural sources. In this regard, plants, and some microbial species, due to their composition, ecology, phytochemical, and ethnopharmacological properties, play a significant role. Accordingly, a series of plant-derived bioactive compounds are in the clinical development phase against cancer. The present review highlights the significance of several medicinal plants, plant extracts, and their bioactive compounds for their potential anticancer activities. The presented results can be useful for researchers in developing novel anticancer drugs, public health specialists, and for the public in general.

**Abstract:**

Cancer is one of the major deadly diseases globally. The alarming rise in the mortality rate due to this disease attracks attention towards discovering potent anticancer agents to overcome its mortality rate. The discovery of novel and effective anticancer agents from natural sources has been the main point of interest in pharmaceutical research because of attractive natural therapeutic agents with an immense chemical diversity in species of animals, plants, and microorganisms. More than 60% of contemporary anticancer drugs, in one form or another, have originated from natural sources. Plants and microbial species are chosen based on their composition, ecology, phytochemical, and ethnopharmacological properties. Plants and their derivatives have played a significant role in producing effective anticancer agents. Some plant derivatives include vincristine, vinblastine, irinotecan, topotecan, etoposide, podophyllotoxin, and paclitaxel. Based on their particular activity, a number of other plant-derived bioactive compounds are in the clinical development phase against cancer, such as gimatecan, elomotecan, etc. Additionally, the conjugation of natural compounds with anti-cancerous drugs, or some polymeric carriers particularly targeted to epitopes on the site of interest to tumors, can generate effective targeted treatment therapies. Cognizance from such pharmaceutical research studies would yield alternative drug development strategies through natural sources which could be economical, more reliable, and safe to use.

## 1. Introduction

Cancer is the anomalous growth of cells in the body; it is the leading cause of death and is also known as the biggest public health burden [1]. Cancer cells can also attack and damage the body’s normal cells [2]. Millions of people have died due to four common types of cancers every year, including breast, lung, prostate, and rectum/colon cancer with an unknown etiology. The present tenet indicates a conspicuous difference between cancer chemotherapy and chemoprevention. Cancer chemotherapy is the control of the developed disease, while cancer chemoprevention is the phenomenon of a carcinogenesis intervention by blocking the agents of the induction of the neoplastic process or averting the processing of transformed cells to the malignant phenotype using suppressing agents. Cancer chemoprevention may also implicate the reversal of the progression of cancer cells [3].

**Figure 1:**
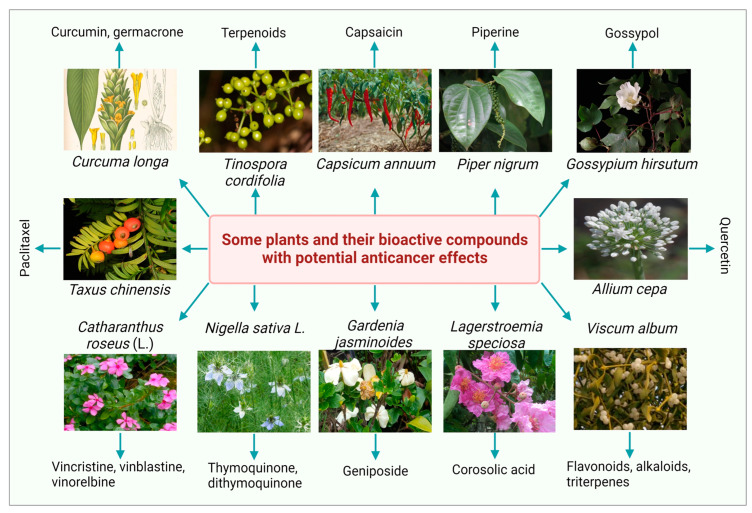
Some medicinal plants and their bioactive compounds having potential anticancer properties.

The investigation of anticancer agents through natural sources dates back to about 1550 BC. However, the scientific exploration of this research is very recent and originated in the 1950s with the generation of majorly found plant-derived anticancer agents, including vinca alkaloid analogs, camptothecin derivatives, podophyllotoxin derivatives, and taxol semi-synthetic analogs which are clinically helpful anticancer therapeutic drugs (Figure 1) [4,5]. Over 180,000 microbial-derived anticancer agents, 16,000 marine-derived organisms, and 114,000 plant-derived compounds were screened by the US National Cancer Institute (NCI) for their anti-cancerous activity from the 1960s to the 1980s [6]. Plant-based drug development also provided a platform for synthesizing efficient and safe anti-tumor drugs through the complete cognizance of a synergistic relation between numerous components of anti-tumor herbs [7,8]. According to the WHOs estimation, approximately 80% of African and Asian countries rely on traditional medicines for fundamental health care. A neoteric study shows that approximately more than 60% of patients use herbs or vitamins as cancer therapy [9,10]. Herbal remedies are among the most favored form of traditional medicine and are tremendously profit-making at the international commercial level. By the 2050s, the worldwide herbal medicine market is expected to hit USD 5 trillion [11].

Natural products provide a sustainable source with a considerable efficacy to treat and overcome several disorders and fatal diseases, including cancer. In the last time period, the role of the bioactive compound and natural products, as a source of anticancer drugs, has been marked within a collaborative, integrated, and multidisciplinary approach. Plants have long been known for having medicinal effects since aeon [12,13,14,15,16]. More than 50% of modern clinical drugs are of a natural source origin and have the capability to treat cancer cells [17]. A neoteric study shows that approximately more than 60% of patients use herbs or vitamins as cancer therapy. The ability of natural sources as anticancer agents were identified in the 1950s by the US National Cancer Institute and contributed to finding new naturally existing anti-tumor agents [18]. Plant-based drug development needs a specific production strategy with optimized environmental conditions and nutrient availability. In 1998, Sohn et al. estimated that an extraction from 10,000 kg of the bark of yew trees is required to produce 1 kg of taxol. The production of 25 kg of taxol required 38,000 yew trees for the treatment of 12,000 cancer patients [19]. The plant collection for finding anticancer agents ended in 1982, but in 1986, the generation of new screening strategies led to the amelioration of plants and the collection of other organisms mainly focused on the sub-tropical and tropical zones of the world. Hartwell listed more than 3000 plants in his review against cancer treatment [20]. Various anti-cancerous drugs are available to treat cancer, but they also exhibit toxic effects that limit their use [18,21]. Because of the severe side effects of radiotherapy and chemotherapy and the high mortality rate, recent research revolves around the need to design appropriate chemotherapy for cancer treatment without side effects [21,22]. Biodiversity has been determined to be a significant source of remarkable anticancer agents until now [23,24,25,26,27,28].

A significant investigation is devoted to finding more effective treatments with minimum undesirable toxic effects. However, many anti-tumor agents exhibit a restricted therapeutic window due to a lack of specificity of against cancer cells [29,30]. The ultimate objective of a cancer treatment is the generation of safe and effective drugs that can particularly kill malignant cancer cells or make them benign cancer cells without killing normal cells [31]. This review aims to highlight the significance of several medicinal plants, plant extracts, and their bioactive compounds with potential anticancer activities to facilitate the researchers to develop novel anticancer drugs providing some considerable benefits, such as highly selective and significant anticancer activity, less to no toxicity, low side effects, being economic and eco-friendly, as well as providing a cancer preventing role via an increasing immunity [32,33,34].

## 2. Methodology

A descriptive review was conducted in order to evaluate the anticancer potential of several natural plants and their bioactive compounds. The articles documented on anticancer, chemopreventive, and cytotoxic effects with experimental investigations (such as in vitro, in vivo, and clinical trials) were critically evaluated. The bibliographic material was collected using the Google Scholar, ScienceDirect, Web of Science, and PubMed databases. Several search terms such as “natural plants”, “medicinal plants”, “bioactive compounds”, “phytoconstituents”, “phytochemicals”, “plant extracts”, “anticancer agents”, “plant-derived therapeutics”, “cytoxicity analysis”, “antiproliferative”, and “apoptotic effects” were used to collect literature in a variety of combination forms. The generated articles were screened in order to fulfill the desired selection criteria by evaluating their titles and further abstract summaries. The irrelevant articles, like those with any other language except English, or articles with insignificant outcomes, aiming to evaluate natural plant products or their bioactive compounds as effective anticancer agents treating different types of cancers, were excluded from the selected data. Moreover, all of the selected publications were exclusively read to gather effective literature based on their potential anticancer effects indicated by experimental investigations.

## 3. Plant-Derived Bioactive Compounds as Anti-Cancerous Agents

Over the last decade, several researchers have investigated the ethnopharmacological and ethnomedicinal properties of numerous plant-derived bioactive compounds and, recently, their antimicrobial and antibiofilm activities [35]. Several in vitro and in vivo experimental investigations revealed the therapeutic significance of numerous phytochemicals (Table 1). Some photos of the most studied plants with a significant anticancer potential with their bioactive compounds are presented in Figure 1. The most common plant-derived anti-cancerous agents include vinca alkaloids and their derivatives, camptothecin and its derivatives, podophyllotoxin and its semi-synthetic analogs, and terpenes.

### 3.1. Vinca Alkaloids and Their Derivatives

**Figure 2:**
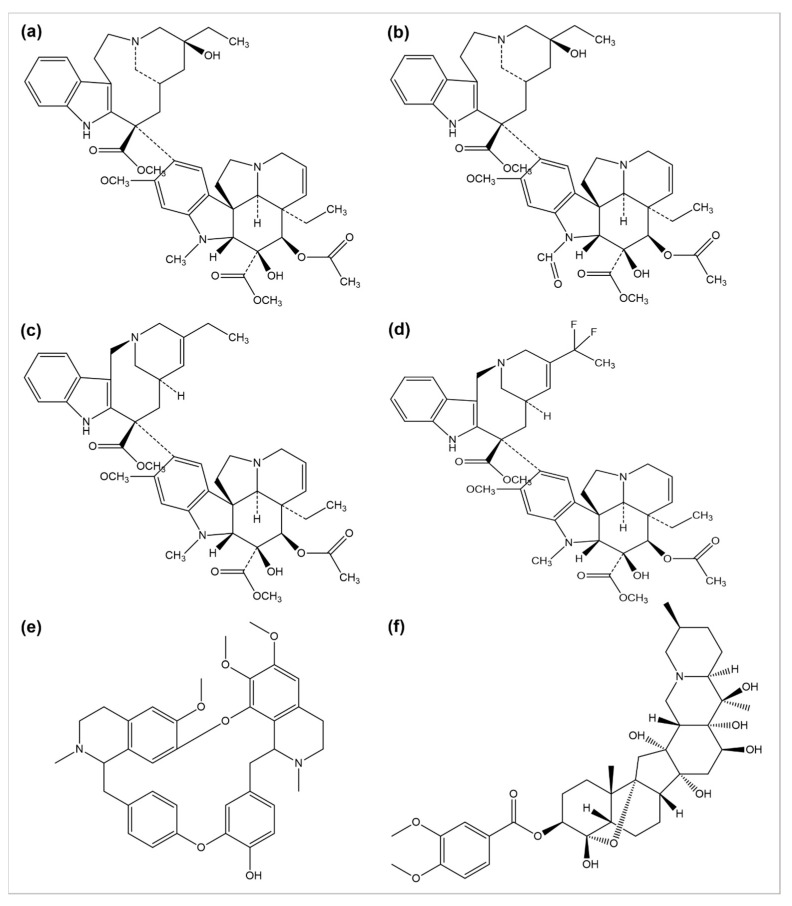
Chemical structures of (**a**) vinblastine, (**b**) vincristine, (**c**) vinorelbine, (**d**) vinflunine, (**e**) berbamine, and (**f**) veratridine.

The use of plants as anticancer agents was established with two alkaloids’ isolation, vincristine, and vinblastine, using *Catharanthus roseus* and *Madagascar periwinkle* [93]. These drugs have been clinically used in oncology for about 50 years. They perform their function by blocking the polymerization phenomenon of tubulin molecules, averting the mitotic spindle formation, and resulting in apoptosis or metaphase arrest [94]. Several anticancer drugs, such as vincristine, vinblastine, vinorelbine, vinflunine, veratridine, and berbamine, are plant-derived natural alkaloids (Figure 2).

A number of semi-synthetic analogs of these two alkaloid drugs have been produced. Vindestine was produced by the replacement of the C acetyl group with an amino group in vinblastine [95], primarily applied for the treatment of acute lymphocytic leukemia (ALL) and rarely prescribed for chronic myelocytic leukemia (CML), breast cancer, non-small cell lungs cancer (NSCLC), colorectal cancer, and renal cancer treatment. Vinorelbine (also known as navelbine) is another semi-synthetic analog of vinblastine synthesized by shortening one carbon from the indole ring linking the bridge to piperidine nitrogen, resulting in a water elimination from the piperidine ring, and was approved in 1989 in France for the treatment of NSCLC under the brand name Navelbine. Vinflunine, a dihydrofluoro semi-synthetic analog of vinorelbine, is used as the second line of treatment in metastatic urothelial cancer. It was approved in 2009 by the European medical agency [96,97]. Alike other semi-synthetic analogs of vinca alkaloids, vinflunine also attaches to tubulin molecules resulting in the inhibition of microtubule polymerization and the formation of tubulins para crystals [98,99,100,101].

Cao et al. investigated the anticancer effects of 13 isoquinoline alkaloids extracted from *Hylomecon japonica* on MCF-7 breast cancer cells. Among these 13 alkaloids, 6,10-dimethoxydihydrochelerythrine, 6S/R-acroleinyl-dihydrochelerythrine, 9-methoxy-10-hydroxy-norchelerythrine, 10-methoxy boconoline, 6-methoxydihydrosanguinarine, dihydrosanguinaline, and 6-acetaldehyde-dihydrochelerythrine exhibited a significant inhibitory potential with an IC_50_ of ˂20 μM on MCF-7 cells [102]. Freeling et al. determined the tumor suppression potential of the plant-based alkaloid veratridine (VTD). VTD activates the expression of UBXN2A (an anti-tumor protein) by deactivating a dominant protein, mortalin, involved in the development of cancer [103]. Liu et al. evaluated the antiproliferative and anti-migratory effect of the alkaloid berbamine. Berbamine suppressed the growth of negative breast cancer cells by regulating the PI3K/Akt/mTOR and PI3K/Akt/MDM2/p53 pathways [104]. Esnaashari et al. investigated the synergistic effect of the alkaloid doxorubicin (DOX) with noscapine-loaded polymeric nanoparticles (NOS-NPs) for breast cancer treatment. The anticancer potential of NOS-NPs combined with DOX and alone was evaluated against 4T1 breast cancer cells (in vitro) and mice (in vivo). The NOS-NPs, in combination with DOX, significantly showed a 68.50% inhibition against the growth of breast cancer. The DOX and NOS-NPs alone exhibited a 32 to 55.10% inhibition, respectively [105].

### 3.2. Camptothecin and Its Derivatives

**Figure 3:**
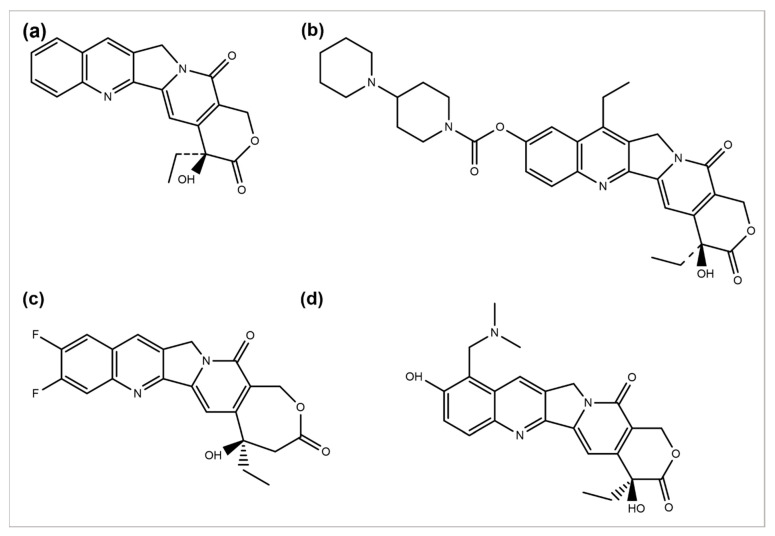
Chemical structures of (**a**) camptothecin, (**b**) irinotecan, (**c**) diflomotecan, and (**d**) topotecan.

*Camptotheca acuminata* plant species are a source of the anticancer agent camptothecin (CPT), a quinoline alkaloid that acts by inhibiting the activity of topoisomerase-I, causing DNA damage and, ultimately, cell death [106]. Because of its severe toxicity and low aqueous solubility, it was terminated from clinical trials. Several CPT derivatives are developed and approved for clinical use to combat these limitations. Some of the CPT derivatives are irinotecan, belotecan, and topotecan, which actively inhibit DNA topoisomerase–I, an enzyme involved in DNA replication and transcription (Figure 3a,b,d) [107]. 9-aminocamptothecin (9-AC) is another CPT semi-synthetic derivative that exhibited a sound activity effect pre-clinical analysis but has not shown clinically effective anticancer activity hitherto.

In 1993, 9-AC entered in phase-I trials and revealed the dose-dependent phenomenon of myelosuppression as a major toxic effect of the respective drug. Subsequently, in phase-II trials, the drug was found to be active against malignant and ovarian lymphoma and inactive against colon or lung cancer. Consequently, in 1999, it was terminated from any further development [108]. However, some phase-I or II trials have been reviewed to predict its efficacy, safety, and tolerability separately or in combination with some other analogs [109]. Several drugs such as diflomotecan (for advanced solid tumors treatment at phase I) (Figure 2c) [110], gimatecan (for advanced solid tumors treatment at phase I) [111], (for recurrent ovarian, peritoneal, or fallopian tumor treatment at phase II) [112], elomotecan (for advanced solid tumors treatment at phase I) [113], and EZN-2208 (for advanced malignancies treatment) [114] have been reported as clinical trial-based studies.

### 3.3. Podophyllotoxin and Its Semi-Synthetic Analogs

**Figure 4:**
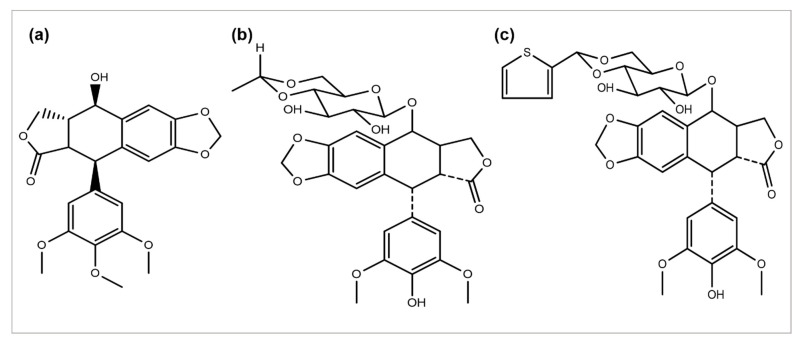
Chemical structures of (**a**) podophyllotoxin, (**b**) etoposide, and (**c**) teniposide.

*Podophyllum peltatum* plant is an important source of the anticancer compound Podophyllotoxin and has two key analogs, Teniposide and Etoposide (Figure 4) [115], which are useful in the treatment of different types of cancer acts by inhibiting the function of the topoisomerase II enzyme [5]. The above two analogs combat some problems and issues, such as a metabolic inactivation, poor water solubility, and acquired drug resistance. The improved efficacy and potency led to the development of some semi-synthetic derivatives, including azatoxin, NK-611, Top-53, tafluposide, GL-331, and etoposide phosphate, either as clinical drugs or new trial candidates for cancer treatment [116].

### 3.4. Taxane Diterpenoids

**Figure 5:**
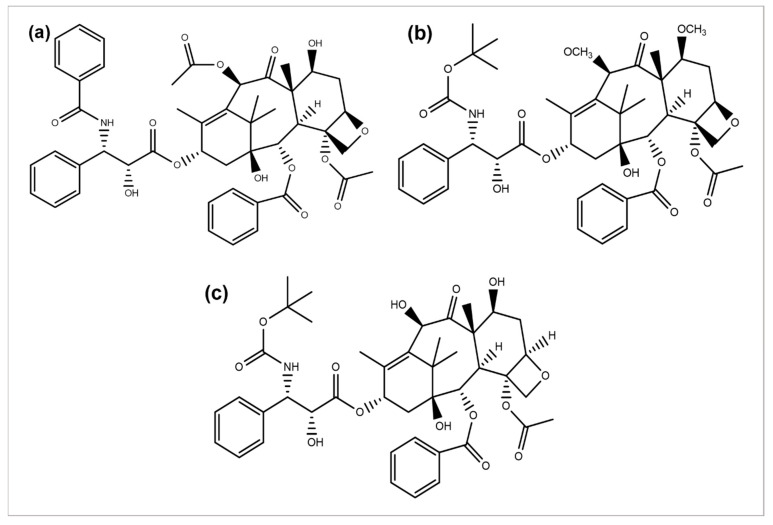
Chemical structures of (**a**) paclitaxel, (**b**) cabazitaxel, and (**c**) docetaxel.

Paclitaxel discovery from the bark extract of the Yew tree further provided evidence for a successful drug discovery through natural products. Taxol was the first compound discovered for a microtubule synthesis promotion. It has been known to be used in treating several types of cancers, particularly breast, ovarian, and NSCLC [117]. A wide range of its derivatives has been produced (Figure 5). Docetaxel was the first to be clinically used with significant clinical activity against different tumors [118,119]. Both of the authorized taxane drugs, paclitaxel and docetaxel, still have limitations of use, and the researchers are trying to overcome their side effects by synthesizing the modified derivatives. Alterations in their structures has led to the discovery of new agents with a diminished toxicity, enhanced solubility, and refined cytotoxicity. The restricted ability of docetaxel and paclitaxel to cross the blood–brain barrier is concluded to be generated by the P-glycoprotein efflux pump tremendously expressed in the BBB [120,121,122]. In 2010, another FDA-approved taxane derivative, Cabazitaxel, was established in combination with prednisone for treating hormone-refractory prostate and prostate cancers. Cabazitaxel suppresses the proliferation of cancer cells by stabilizing tubulin and inhibiting the depolymerization of microtubules [123]. Nanoparticle formulations are also being applied to obtain better results. Abraxane is the albumin-bound nanoparticle-based formulation of paclitaxel free of any solvent, which acts as a mitotic inhibitor, and shows that it can have dramatically improved effects. New taxanes are also being developed to improve the therapeutic effect and pharmacology and replace docetaxel and paclitaxel which are currently used for the treatment of NSCLC [124].

## 4. Some Other Plant-Derived Anticancer Agents

Omacetaxine mepesuccinate is an alkaloid anticancer agent extracted from *Cephalotaxus harringtonia* and approved by the FDA; it is a translation process inhibitor. Omacetaxine inhibits the translation of proteins by inhibiting the elongation of the protein synthesis process by interfering the A-site and averting the correct amino acid positioning to aminoacyl tRNA [125]. Ingenol mebutate is found in the plant sap of *Euphorbia peplus*, and in January 2012, it was approved by the US FDA and EMA in gel form for the acid keratosis treatment. This compound is an ester of angelic acid and diterpene [126]. Betulinic acid is a secondary metabolite of *Betula* species from the Betulaceae family, occurring in natural form as pentacyclic triterpenoid. It was isolated from the *Zizyphus* plant species, like *Mauritiana oenoplia* and *Mauritiana rugose* [127,128], exhibiting numerous biological activities such as anti-oxidant, antibacterial, anti-inflammatory, anti-retroviral, and anti-HIV properties [129,130]. Dai et al. conducted the in vivo and in vitro studies to determine the anticancer potential of *Taxus chinensis* var. *mairei* (TC) against lung cancer. The aqueous extract of TC showed a significant anticancer effect by degrading CD47 with less toxicity [131]. Wu et al. investigated the antitumor efficacy of a polysaccharide (CPTC-2) isolated from *Taxus chinensis* var. *mairei* against gastric cell lines (SGC-7901). The outcomes obtained by the flow cytometry and MTS assay revealed significant antitumor activity in a dose-dependent way [132].

Flavopiridol is a synthetic compound having an identical structural similarity with rohitukine extracted from an Indian indigenous plant, *Dysoxylum binectariferum* [133]. It is known for targeting the cyclin-dependent kinase activity, including the cyclin T complex or CDK9, suppressing the regulation of Mc1-1 and anti-apoptotic proteins, and inducing changes in the permeability of mitochondria [134,135,136]. It is also known as the first potential inhibitor of cyclin-dependent kinases to gain clinical trials [137]. *Curcuma longa* plant, also known as turmeric, is derived from the polyphenol Curcumin and has a wide range of therapeutic properties, including anti-inflammatory, analgesic, antiseptic, and antioxidant activity [138]. Turmeric plants contain some compounds called curcuminoids containing curcumin, bisdemethoxycurcumin, and demethoxicurcumin. Of all curcuminoids, curcumin has a significant therapeutic effect [139]. It has also been found to have anticancer activity by affecting the biological pathways connected with oncogene expression, mutagenesis, metastasis, apoptosis, and the regulation of the cell cycle [139].

## 5. Mushrooms as a Source of Anticancer Agents

Mushrooms carrying medicinal properties have an established history as a traditional medicine. Mushroom-derived therapeutic substances can function in human bodies and are progressively being grown [140]. Numerous traditionally utilized mushrooms from the genera of Trametes, Ganoderma, Auricularia, Tremella, and Flammulina are known to have remarkable medicinal properties [141]. Many medicinal properties of mushrooms have dragged researchers’ attention to explore the finding of new mushrooms and their important metabolites, bearing profound anticancer and antioxidant properties [142]. In 1975, Lucas and his fellows first described the anticancer activity of higher Basidiomycetes [141]. The considerable physiological properties and pharmacological efficacy of medicinal mushrooms are of a homeostasis maintenance, the enhancement of the immune response (bioregulation), the biorhythm regulation, the prevention and cure of different diseases, and the amelioration of human health from life-threatening diseases such as heart diseases, cancer, and cerebral stroke, etc. Mushrooms are also known to have antibacterial, anti-mycotic, antiviral, anti-tumor, anti-inflammatory, antithrombotic, antidiabetic, hypotensive, and hypolipidemic activities [143]. Rutckeviski et al. investigated the synergistic effect of *Agaricus bisporus* extract β-(1→6)-d-glucan in combination with doxorubicin against breast cancer cells (MDA-MB-231). The outcomes exhibited a synergistic effect of doxorubicin and *A. bisporus*, which decreased the 31% viability of the tumor cells. Moreover, a β-(1→6)-d-glucan treatment combined with doxorubicin enhanced the sensitivity of MDA-MB-231 to doxorubicin [144].

Yoon et al. investigated the anti-tumor effect of the derivatives of adenosine isolated from *Cordyceps militaris* against ovarian cancer cells. The outcomes revealed autophagic death, mediated by adenosine derivatives in ovarian cancer cells by the ENT1-AMPK-mTOR pathway [145]. Cen et al. evaluated the anticancer potential of *Ganoderma lucidum* against ovarian cancer. The obtained results exhibited the reactive-induced species-induced activation of the ERK pathway [146]. Thimmaraju et al. studied the anti-tumor effect of polysaccharide (HUP-2) isolated from *Hypsizygus ulmarius*. HUP-2 was isolated using the hot water extraction method. HUP-2 revealed considerable cytotoxicity and inhibition against PC3 prostate cells [147]. Fekry et al. determined the anticancer potential of *Pleurotus ostreatus* (selenium enriched mushroom) in colon cancer. The outcomes showed significant anticancer activity by enhancing the production of IL-6 and IL-10, reducing the production of TNF-α and the targeting of the Raf-1 pathway [148]. Meng et al. studied the antitumor effect of water-soluble polysaccharide obtained from *Boletus edulis* (BE) mushroom against breast cancer cells (Ca761, MDA-MB-231) using an MTT assay. The obtained outcomes revealed that BE can significantly induce mitochondrial apoptosis and proliferation inhibition [149]. Another study revealed the potential anticancer effect of the silver nanoparticles (Ag NPs) prepared by *Boletus edulis* and *Coriolus versicolor* mushrooms against colorectal, breast, and hepatocellular carcinoma cells (HT-29, MCF-7, and HUH-7 cells, respectively). The outcomes showed significant anticancer activity by inhibiting proliferation and ROS-generated apoptosis [150].

Some examples of significant medicinal mushrooms with anti-tumor properties are given in Table 2.

Several researchers have reported the anti-tumor potentials of different mushroom species and reduced the adverse effects like anemia, nausea, insomnia, drug resistance, and bone marrow suppression after radiation and chemotherapy [170]. Additionally, some potential mushroom species have been evaluated in clinical trials (Table 3) [171].

A medicinally reported mushroom, *Agaricus blazei,* has been revealed to possess significant anti-tumor activity by an enlarged number of T-regulatory and plasmacytoid dendritic cells and an enhanced level of human leukocyte, immunoglobulin, and killer-immunoglobulin receptor genes [172]. Misgiati et al. isolated ergosterol using n-hexane extracts prepared with *Agaricus blazei Murill* mushroom. The anticancer activity of the ergosterol using an MTT assay was identified against MCF-7 cells. The outcomes showed significant anticancer activity with an IC50 of 43.10 µg/mL by inhibiting the cell cycle and inducing apoptosis [173]. Sun et al. extracted an RNA-protein complex (FA-2-b-B) using *Agaricus blazei Murill* to evaluate the anticancer potential against chronic myeloid leukemia. The outcomes exhibited that the FA-2-b-B protein complex has a significant proapoptotic and antiproliferative effect, suggesting that the intake of *Agaricus blazei Murill* may provide an effective alternative approach for the management and treatment of chronic myeloid leukemia [174].

Jeitler et al. reported a considerable decrease in anorexia over time after 6 cycles of *Agaricus sylvaticus* combined with chemotherapy; simultaneously, some side effects such as anorexia, vomiting, diarrhea, nausea, and constipation were observed in the placebo group [175]. *Coriolus versicolor* was used for the treatment of hepatocellular carcinoma patients that are mostly inoperable. No difference was observed with the *Coriolus versicolor* treatment compared to the placebo group, possessing an improved quality of life compared to the placebo on the treatment. This study suggested using a supplementation for palliative care [176].

The *Agaricus bisporus* (white button mushroom) powder was used for the treatment of prostate cancer. A continuous rise in the levels of a prostate-specific antigen (PSA) was observed in patients with prostate cancer; an escalation in the PSA level may direct the recurrence of the disease. The results showed a therapy-induced drop in myeloid-derived suppressor cells, and patients with partial and complete responses exhibited increased baseline interleukin-15 levels compared to the non-respondents [177]. Torkelson et al. conducted a phase-I trial using *Trametes versicolor* against immune-compromised patients of breast cancer. They reported an improved immune status with the enhanced activity of natural killer cells, lymphocyte counts, and a dose-dependent increase in CD^8+^ T-cells and CD^19+^ B-cells. It can be suggested that a *Trametes versicolor* treatment can be used to improve the immunity levels in the immune-compromised patients of breast cancer [178].

## 6. Conclusions

The extracts or bioactive compounds from plants and fungi exhibit several mechanisms with anti-tumor effects. The mushroom and plant-derived bioactive compounds may regulate or activate the immune system, by disturbing the immune cell’s maturation, differentiation, and proliferation mechanisms, thus inhibiting the growth of cancer cells. Plant-derived compounds may not directly be used as drugs, but they encouraged the researchers to design and develop novel anticancer agents. A thorough understanding of the mechanisms of action of plant and mushroom-derived bioactive compounds with anticancer properties is essentially required for cancer treatment, providing cancer patients with an improved quality of life. However, the clinical studies of numerous plant and mushroom-derived bioactive compounds reveal significant anticancer potentials with immunomodulation and the reduced side effects of conventional treatments. Consequently, more clinical studies must be conducted, particularly by applying a prime methodology, standard preparations, large sample sizes, and long-lasting follow-ups. Future studies should also focus on establishing the preventive or defensive aspects of plants and mushrooms for reducing the development of cancers, by including them in one’s daily life for a healthy diet.

## Figures and Tables

**Table 1:** Plant-derived bioactive compounds as anti-cancerous agents along with their modes of action.

Plant Source	Bioactive Compound/ Phytochemical	Cancer Cells Type	Study Type	Mode of Action	Ref
*Aconitum sinomontanum*	Lappaconitine	Liver	In vitro	Downregulation of Bcl-2 and upregulation of P53 and Bax expression	[36]
*Alliaria petilata*	Benzyl isothiocyanate	Colon	In vitro	MAPK and PKC pathways inhibition	[37]
*Allium cepa*	Quercetin	Thyroid	In vitro	Pro—NAG-1/GDF15 pathways upregulation	[38]
*Artemisia annua*	Artemisinin	Breast	In vitro	G2/M (cell cycle) arrest, autophagy, antiproliferative, apoptosis	[39]
*Camellia sinensis*	Catechins	Prostate	In vitro	Increased expression of cytochrome c and decreased expression of B-cell lymphoma-2 induced apoptosis	[40]
*Cannabis sativa*	Cannabinoids	Liver	In vivo	Anti-apoptotic	[41]
*Capsicum annuum*	Capsaicin	Breast	In vitro, in vivo	NF-kB inactivation mediated by the FBI-1 downregulation	[42]
*Capsicum frutescens*	Capsaicin	Pancreatic	In vitro, in vivo	β-catenin/TCF-1 signaling inhibition-mediated apoptosis	[43]
*Carica papaya*	Benzyl isothiocyanate	Pancreatic	In vitro, in vivo	FOXO/PI3K/AKT pathways-mediated tumor apoptosis	[44]
*Chamaemelum nobile*	Phenolic compounds	Breast	In vitro	Mitochondrial pathway activation-induced apoptosis	[45]
*Citrus limon*	Hesperidin	Breast	In vitro	NF-kB and Akt downregulation-mediated PD-L1 expression inhibition	[46]
		Prostate	In vitro	ROS-induced apoptosis	[47]
*Crocus sativus*	Safranal	Prostate	In vitro, in vivo	Downregulation of NF-kB and AKT signaling pathways	[48]
*Cucumis sativus*	Cucurbitacin B	Neuroblastoma	In vitro	MAPKs- and JAK2/STAT3-mediated apoptosis	[49]
	Cucurbitacin B (in combination with gefitinib)	Colorectal	In vitro	JAK/STAT and EGFR-induced apoptosis	[50]
*Denrobium chrysotoxum*	Erianin	Breast	In vitro	PI3K/Akt pathway activation	[51]
*Eclipta alba*	Luteolin	Breast	In vitro, in vivo	Intrinsic apoptotic pathway activation	[52]
*Galanthus nivalis*	Gallic acid	Colon	In vitro, in vivo	EGFR and SRC phosphorylation inhibition	[53]
		Liver	In vitro	Wnt/β-catenin pathway suppression	[54]
*Glycyrrhiza glabra*	Licochalcone A	Lung	In vitro	JNK suppression, P38 and ERK activation	[55]
*Gossypium hirsutum*	Gossypol	Skin	In vitro	Mitochondrial apoptosis	[56]
		Cervical	In vitro, in vivo	FAK pathway inhibition and TGF-β1-mediated EMT reversal	[57]
		Colon	In vitro	Downregulation of FAS, CLAUDIN1, GAPDH, ELK1, ZFAND5, IL2, and IL8 expression	[58]
*Lagerstroemia speciosa*	Corosolic acid	Bladder	In vitro, in vivo	SQSTM1/P62, UBB, and NBR upregulation	[59]
		Liver	Ex vivo, in vitro, in vivo	YAP/CDK19/O-GlcNAcylation inactivation	[60]
		Colon	In vitro, in vivo	HER2 and HER3 heterodimerization inhibition	[61]
*Mortonia greggii*	Pristimerin	Lung	In vitro	MMP2 and integrin β1 expression downregulation	[62]
*Myrica nagi*	Myricetin	Lung	In vitro	FAK-ERK pathway inhibition	[63]
*Nelumbo nucifera*	Hyperoside, rutin	Colon	In vitro	Mitochondrial pathway activation-induced apoptosis	[64]
*Panax ginseng*	Ginsenosides	Breast	In vitro, in vivo	VEGF-R2 pathway inhibition correlated with anti-angiogenesis	[65]
				ROS generation, mitochondrial dysfunction, apoptosis	[66]
*Papaver somniferum*	Noscapine	Colon	In vitro	AKT/PI3K/mTOR pathway inhibition	[67]
*Perovskia abrotanoides*	Tanshinones	Hella cell lines	In vitro	Antiproliferative, apoptosis	[68]
*Piper longum*	Piperlongumine	Prostate	In vitro	DNA damage-mediated proliferation inhibition	[69]
		Lung	In vitro, in vivo	mTOR/AKT/PI3K pathway inhibition-induced apoptosis	[70]
*Piper nigrum*	Piperine	Colon	In vitro	Wnt/β-catenin pathway suppression	[71]
*Polygonum cuspidatum*	Pterostilbene	Colon	In vitro, in vivo	DNA repairing by Top1/Tdp1 pathway	[72]
*Pongamiopsis pervilleana*	Epipervilline	Ovarian	In vitro	Antiproliferative	[73]
*Pueraria radix*	Puerarin	Prostate	In vitro	Keap1/Nrf2/Are pathway inhibition	[74]
*Quercus alba*	Quercetin	Prostate	In vitro	ROS modulation, AKT/NF-kB pathway activation	[75]
*Reseda luteola*	Luteolin	Lung	In vitro	FAK-Src signaling inhibition	[76]
*Rheum palmatum*	Emodin	Lung	In vitro	HAS2-HACD44/RHAMM signaling pathway suppression	[77]
*Ruta graveolens*	Psoralens	Breast, colon, and prostate	In vitro	Inhibits proliferation of cancer cells	[78]
*Salvia involucrate*	Hispidulin	Lung	In vitro, in vivo	ER stress activation-induced apoptosis	[79]
*Solanum lycopersicum*	Lycopene	Cervical	In vitro	Bcl-2 downregulation and Bax upregulation	[80]
		Lung	In vitro, in vivo	Increase in RARβ protein expression	[81]
		Brain	In vitro	Caspases activation	[82]
	Lycopene (in combination with quinacrine)	Breast	In vitro	Wnt-TCF signaling inhibition	[83]
*Sophora flavescens*	Matrine	Liver	In vitro, in vivo	HOXD3 and circ-0027345 downregulation and miR-345-5p upregulation	[84]
*Spinacia oleracea*	Kaempferol	Pancreatic	In vitro	ROS-induced Akt/mTOR signaling inactivation	[85]
		Breast	In vitro	Upregulation of caspase-3 and -9 and H2AX expression	[86]
*Trianthema portulacastrum*	Ecdysterone	Breast	In vivo	β-catenin/Wnt signaling inhibition inducing pro-apoptotic and antiproliferative effects	[87]
*Vitis vinifera*	Resveratrol	Breast	In vitro	Cell cycle inhibition, apoptosis	[88]
		Osteosarcoma	In vitro, in vivo	STAT3 pathway and cell cycle inhibition	[89]
		Colorectal	In vitro	NF-kB pathway inhibition, apoptosis	[90]
*Zingiber officinale*	Gingerol	Lung	In vitro, in vivo	A549 cells death by iron accumulation, USP14 expression inhibition	[91]
		Breast	In vitro	ROS generation, activation of p53 expression mediated apoptosis,	[92]

**Table 2:** Some mushroom species, along with their anti-tumor activities.

Mushroom Specie	Extract/Bioactive Compound	Cancer Type	Study Type	Outcomes	Ref
*Boletus edulis*	Polysaccharide	Breast	In vitro (Ca761, MDA-MB231 cells)	Proliferation inhibition and mitochondrial apoptosis.	[149]
	Antitumor protein	Non-small cell lung cancer	In vivo and in vitro (A549 cells)	Cell cycle arrest at G1 phase and apoptosis.	[151,152]
*Boletus edulis,* *Coriolus versicolor*	Silver nanoparticles	Colorectal, breast and hepatocellular carcinoma	In vitro (HT-29, MCF-7, HUH-7 cells)	Cell viability inhibition by proliferation inhibition and ROS-generated apoptosis.	[150]
*Cantharellus cibarius, Coprinus comatus, Lactarius deliciosus, Lycoperdon perlatum*	Ethanol and water extracts	Glioblastoma	In vitro (LN-18, U87MG cells)	Proliferation inhibition, cell cycle arrest at G1 and G2/M phase induced apoptosis, metallo-proteinases inactivation.	[153]
*Innotus obliquus*	Hot water extract	Breast	In vivo (mice)	Anticancer activity by innate immunity activation	[154]
*Ganoderma lucidum*	Spore oil	Breast	In vitro (MDA-MB231 cells), in vivo (mice)	Mitochondrial apoptosis	[155]
*Pleurotus highking*	Purified fraction III	Breast	In vitro (HCC-1937, MDA-MB-231 cells)	Akt signaling suppression induced proliferation and migration inhibition	[156]
*Lignosus tigris*	Cold water extract	Breast	In vitro (MCF-7 cells), in vivo (mice)	Proliferation inhibition, signaling pathways induced apoptosis, and tumor growth inhibition.	[157]
*Sarcodon imbricatus*	Water extract	Breast	In vitro, in vivo (MCF-7, 4T1 cells)	Tumor growth inhibition by inhibiting migration and invasion of tumor cells and immunomodulatory activity.	[158]
*Taiwanofungus camphoratus*	Mycelia broth	Adenocarci-noma	In vitro (A549 cells)	Caspase-3 and ROS-induced apoptosis.	[159]
*Ganoderma neojaponicum*	Butanol, chloroform, hexane, and water extracts	Colon	In silico (HT29 and CT 116 cells), in vitro	Proliferation inhibition by apoptosis induction.	[160]
*Hexagonia glabra*	Water, ethyl acetate, and ethanol extract	Cervical cancer	In vitro (CaSki, HeLa, and SiHa cells)	Apoptosis induced by cell cycle arrest at G2/M and increased expression of caspase-3 and -9.	[161]
*Calocyba indica*	Ethanol extract	Pancreatic	In vitro (MIAPaCa2 and PANC-1 cells)	Modulation of p53 and caspase-3 and -9-induced apoptosis and growth inhibition.	[162]
*Fomitopsis pinicola*	Ethanol extract	Prostate cancer	In vivo (Mouse model-PCa cells)	Reduction in tumor growth.	[163]
*Ganoderma lucidum*	Ethanol extract	Hepatocarci-noma	In vitro (SK-Hep1 and QGY7703), in vivo (mice)	Ras/Raf/MEK/ERK pathways inhibition-mediated anticancer activity.	[164]
	Water extract	Glioblastoma	In vitro (GBM8901 and U87MG cells)	Proliferation suppression, cell cycle arrest at S-phase, mitochondrial apoptosis.	[165]
*Ganoderma tsugae*	Ethanol extract	Endometrial	In vitro (KLE, AN3 CA, and HEC-1-A cells)	Proliferation inhibition, G1/S phase cell arrest, Akt signaling pathway inhibition, mitochondrial apoptosis.	[166]
*Heterobasidion annosum*	Methanol extract	Colon	In vivo (mice)	Proliferation suppression by Akt signaling pathway.	[167]
*Termitomyces clypeatus*	Water soluble extract	Astrocytoma	In vitro (HepG2, U373MG, Y-79, MDA-MB-468, U937, HL-60, OAW-42, A549 cells), in vivo (Swiss albino mice)	Anti-tumor effect	[168]
*Antrodia cinnamonea*	Crude ethanol extract	Bladder	In vitro (tsgh-8301, RT4, T24 cells)	Cell death was mediated by anti-migratory activity and down-regulation of cyclin B1 and CDC2	[169]

**Table 3:** List of some significant mushroom species evaluated in clinical trials [171].

Mushroom Specie	Bioactive Compound	Cancer Type	Phase of Study	Status of Study	Identifier Number
*Agaricus bisporus*	Polysaccharides, lectin	Prostate	1b	Completed	NCT00779168
*Agaricus bisporus*	Polysaccharides, lectin	Breast cancer and cancer survivors	1	Completed	NCT00709020
*Agaricus blazei Murill*	Agaricus polysaccharides	Multiple myeloma	2	Completed	NCT00970021
*Lentinula edodes*	Genistein combined polysaccharide	Prostate	-	Completed	NCT00269555
*Lentinula edode*	Arabinoxylan extract combined with *L. edode*	Hepatocellular carcinoma	-	Completed	NCT01018381
*Grifola frondosa*	Polysaccharides	Breast carcinoma, lung neoplasms	1	Completed	NCT02603016
*Omphalotus illudens*	Semi-synthetic derivative of illudin-S	Thyroid	2	Completed	NCT00124527
*Omphalotus illudens*	Semi-synthetic derivative of illudin-S	Recurrent epithelial ovarian cancer	2	Completed	NCT00019552
*Trametes versicolor*	PSK, Krestin, PSP	Breast	1	Completed	NCT00680667

## Data Availability

Data are contained within the article.
